# "The Actor Is Policy": Application of Elite Theory to Explore Actors’ Interests and Power Underlying Maternal Health Policies in Uganda, 2000-2015

**DOI:** 10.34172/ijhpm.2020.230

**Published:** 2020-11-23

**Authors:** Moses Mukuru, Suzanne N. Kiwanuka, Lucy Gilson, Maylene Shung-King, Freddie Ssengooba

**Affiliations:** ^1^Department of Health Policy, Planning and Management, School of Public Health, College of Health Sciences, Makerere University, Kampala, Uganda.; ^2^Health Policy and Systems Division, School of Public Health and Family Medicine, University of Cape Town, Cape Town, South Africa.; ^3^Department of Global Health and Development, London School of Hygiene and Tropical Medicine, London, UK.

**Keywords:** Policy Elites, Millennium Development Goals, Maternal Health, Uganda

## Abstract

**Background:** The persistence of high maternal mortality and consistent failure in low- and middle-income countries to achieve global targets such as Millennium Development Goal five (MDG 5) is usually explained from epidemiological, interventional and health systems perspectives. The role of policy elites and their interests remains inadequately explored in this debate. This study examined elites and how their interests drove maternal health policies and actions in ways that could explain policy failure for MDG 5 in Uganda.

**Methods:** We conducted a retrospective qualitative study of Uganda’s maternal health policies from 2000 to 2015 (MDG period). Thirty key informant interviews and 2 focus group discussions (FGDs) were conducted with national policy-makers, who directly participated in the formulation of Uganda’s maternal health policies during the MDG period. We reviewed 9 National Maternal Health Policy documents. Data were analysed inductively using elite theory.

**Results:** Maternal health policies were mainly driven by a small elite group comprised of Senior Ministry of Health (MoH) officials, some members of cabinet and health development partners (HDPs) who wielded more power than other actors. The resulting policies often appeared to be skewed towards elites’ personal political and economic interests, rather than maternal mortality reduction. For a few, however, interests aligned with reducing maternal mortality. Since complying with the government policy-making processes would have exposed elites’ personal interests, they mainly drafted policies as service standards and programme documents to bypass the formal policy process.

**Conclusion:** Uganda’s maternal health policies were mainly influenced by the elites’ personal interests rather than by the goal of reducing maternal mortality. This was enabled by the formal guidance for policy-making which gives elites control over the policy process. Accelerating maternal mortality reduction will require re-engineering the policy process to prevent public officials from infusing policies with their interests, and enable percolation of ideas from the public and frontline.

## Background

Elite domination and influence over the outcomes of policy processes is widely recognised in public policy literature.^1-3^ Based on our review of literature on policy elites, we describe them as an organized group of actors defined by combinations of skills, access to vital information, social status, economic resources and institutional positions, who come together through common backgrounds, coinciding interests and social interactions.^4-6^ Their decisions regarding a perceived public problem constitute policy.^6,7^ For this reason, elite interests in the policy process have come under scrutiny across sectors.^4^

During the Millennium Development Goal (MDG) period (2000-2015), Uganda put in place several policies addressing MDG 5 concerning maternal health. Despite 15 years of sustained effort, high maternal mortality persisted. By the close of the MDG period, Uganda’s maternal mortality ratio was 368, below the target of 131, deaths per 100000 live births.^8^ Many studies examining performance of maternal health policies have paid significant attention to social determinants of health, epidemiological and health system factors, and effectiveness of interventions.^9-12^ Limited research has been carried out on decision-making, and particularly on the influence of actors and their interests over the performance of maternal health policies. A review published in 2013 highlighted as a major limitation of the MDG framework, the infusion of vested interests into the MDGs by a small group of powerful global actors during the decision-making process.^13^ Another study carried out in 2014 suggests that Uganda’s health policies in the MDG period were influenced by a small group of powerful global and national elites with vested interests.^2^ This study builds on these works to advance our understanding of the exercise of power by policy elites to advance their interests in the policy process. We do so by providing an empirical account of the interests of policy elites and how they exerted influence over Uganda’s maternal health policies during the MDG period. We answer 2 questions: (1) what was the composition and power distribution among the elites who participated in the policy processes for maternal health in Uganda during the MDG period? (2) What were their interests and how did they influence Uganda’s maternal health policies in the MDG period?

### An Overview of Uganda’s Policy Process

The framework for policy-making in Uganda in the MDG period can generally be described as centralised. Policy-making was framed as a central government activity by the 1997 local government act.^14^ The processes, structures and actors were elaborated in the 2009 Government of Uganda generic guidelines for policy-making.^15^ In 2013, the health sector also established guidelines to synchronise its policy processes with overall government processes.^16^ Within this legal framework, all government policies are expected to follow the processes described below.

Policy proposals are initiated by the minister of health informed by the ruling party agenda, cabinet and presidential directives, interest groups’ demands, global agendas, routine policy reviews and national development frameworks. This is followed by consultations led by senior Ministry of Health (MoH) technical staff in strategic positions, sometimes assisted by contracted external experts to generate ideas and draft the policy. The progress of the process is dependent on clearance by: (1) the cabinet secretary who ensures that every new policy addresses the priorities of government, (2) the ministers of finance, planning and economic development, and justice and constitutional affairs who issue certificates of financial and legal implications respectively for any policy proposals (these certificates are a legal requirement without which a policy cannot be approved), and (3) the president who clears the policy for no negative implications across government. Other stakeholders within government include actors from the MoH and related sectors of government. It also involves civil society organizations (CSOs), interest groups, academia, professionals and health development partners (HDPs) in the health sector. When ready, the draft policy (cabinet memorandum) is debated and once approved, it is either handed to the sector for implementation or forwarded into the legislative process if it requires a law or regulations to be operationalised.

### Elite Influence in the Policy Process

We applied elite theory as an analytic framework to explore the interests of policy-makers/actors and how they exerted different forms of power to pursue their interest in the policy process. Our choice of elite theory is based on the nature of Uganda’s policy process which as elaborated, is centralised, and works through structures which are normally occupied by elites. We operationalise elites as an organized group of actors defined by combinations of skills/expertise, access to vital information, control over financial resources and holding strategic institutional positions, who come together through common backgrounds and coinciding interests.^4-6^ These attributes confer power upon policy elites which they apply to exert significant influence on the policy decisions, in contrast to the public’s lack of power.^5^ This study focused on national policy actors. These are individuals who have specific responsibility for developing formal policies in the public or private sectors. Actors also encompass groups, agencies and organizations at national and international levels who seek to influence the formal policy process.^17^

Policy elites are divided into governing and non-governing actors^6,18^ The governing (formal) elites are policy actors who participate in the policy process because they are mandated by their positions in a government agency to make and enforce policies. The non-governing (informal) elites are policy actors who are not part of the official government structures and have their own (personal or organisational) strategic interests to protect and promote, but without whom the government would not function well.^18^

In our analysis, we examined elite interests as our central organising concept, operationalised as the preferences pursued by policy elites (governing and non-governing elites) in the policy process.^19^ Elite interests vary from self-interest, to pragmatism and public interest/altruism; and sometimes are interlinked. Self-interest is reflected in behaviours that seek to maximise personal benefits (economic, political or otherwise), while minimising the personal, negative consequences.^20,21^ Pragmatism is shown when less powerful elites adjust to accommodate the interests of the more powerful, as they try to find practical ways of addressing societal problems. Actors constantly monitor the policy environment and continuously adapt to its pressures as they strive to meet their policy goals.^22^ Public interest/altruism on the other hand entails “the outcomes best serving the long-term survival and well-being of a social collective construed as a public,”^23^ sometimes pursued as a moral obligation to improve welfare.^24^

**Figure 1:**
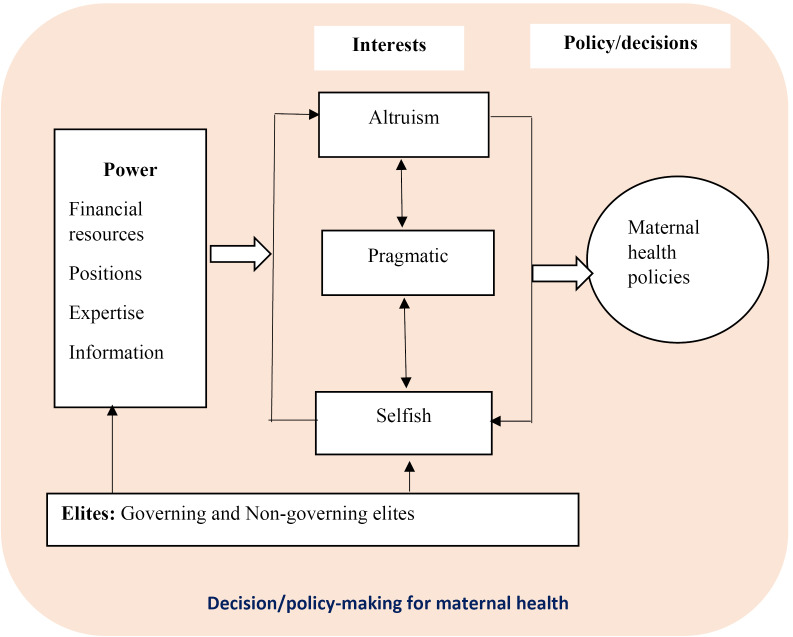
Analytic Framework.

The policy outputs arising from elite decision-making often reflect their interests, because they exert power to influence decisions during the policy process. Power is operationalised as the ability to exercise influence and control over the outcomes (decisions) of the policy process.^18,25^ It is applied in subtle or explicit forms^26,27^ to propel elite interests during decision-making.^25,28^ While power takes different forms, in this paper we have considered access and control over financial resources, control of vital information, skills (especially professional expertise) and positional power (including controlling the structures and managing the policy processes).^29-32^ We concurrently analysed how members of the different elite groups applied power in its different forms to advance their diverse, and sometimes interlinked, interests to influence maternal health policies (see Figure 1).

## Methods

### Study Design

This was a retrospective qualitative study conducted at national level between April and July 2018. It focused on Uganda’s maternal health policies for the period 2000 and 2015 (the MDG period). In this study, it is recognised that maternal health policies are generally considered with policies for reproductive health, basic care package and child health. However, the scope of this analysis was restricted to the maternal health component only. We adopted retrospective policy analysis because it is useful for generating critical lessons from past success and failures to inform future policy reforms.^7^

### Data Collection Methods

**Figure 2:**
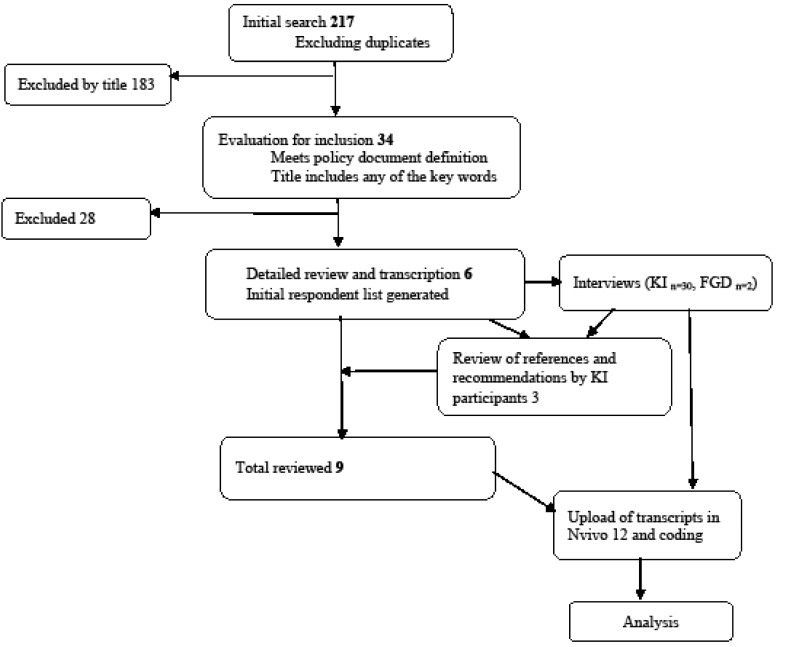
Schema for Document Review and Interviews. Abbreviations: FGD, focus group discussion; KI, key informant.

Data were collected through document review, key informant interviews and focus group discussions (FGDs) (see Figure 2).

We reviewed Government of Uganda maternal health policy documents to understand the actors, the roles they played and how they influenced maternal health policy process. We based our selection of these documents on the World Health Organization (WHO) definition of health policy as “decisions, plans, and actions that are undertaken to achieve specific healthcare goals within a society,”^33^ and Government of Uganda policy-making guidance.^15^ They encompassed policy guidelines and standards, presidential or ministerial directives, programme strategic plans and explicit health policy documents.

A free text search was conducted on the MoH document repository (http://library.health.go.ug/publications). The search terms used were: MDG, Millennium Development Goals, MDG 5, Maternal Health, Women’s Health, Reproductive health, Safe motherhood, Sexual and Reproductive Health, Sexual Reproductive Health and Rights, and, Adolescent Sexual Reproductive Health.

A total of 217 documents were retrieved first scanned by title and then full text. Documents with any of the key search terms in the title (n = 34) were retrieved for evaluation. Inclusion criteria of documents for detailed review were: (1) meeting the qualification of a “policy document” as defined in this study, (2) an explicitly stated goal/objective of reducing maternal mortality anywhere in the content, (3) having a specific component of maternal health and, (4) covering the period 2000-2015. Only 6 documents qualified. Documents without a specific component for maternal health, not for the government of Uganda and not covering the period 2000-2015 were excluded. More documents were identified through review of references (n = 2) and on recommendation by key informants during interviews (n = 1).

In total 9 documents were reviewed (see Table 1). We used a document review template in Microsoft Word to extract the following from each document: (1) actors and the roles they played (these were generally listed in the sections of acknowledgements of the documents), (2) influence of actors (from situational analysis, policy context and rational/justification sections), (3) policy provisions (from sections outlining policy direction/strategic framework/objective/strategies).

**Table 1:** Main Policy Documents That Set Maternal Health Direction in the MDG Period

**Policy Documents**	**Period**	**Justification for Selection**
Health sub-district policy	1999 to date	Bring caesarean section services within 5 km to the families
National health policy	1999-2009	Overall policy framework for the health sector
The national policy guidelines and service standards for reproductive health services	2001-2005	Sets specific goals for RH where maternal health falls
The national policy guidelines and service standards for sexual and reproductive health and rights	2006	Updated specific goals for RH where maternal health falls
Roadmap for accelerating the reduction of maternal and neonatal mortality and morbidity in Uganda	2007-2015	Set specific goals for maternal health for the MDG period
Second national health policy	2010-2020	Updated of the overall policy framework for the health sector
Adolescent health policy guidelines and service standards	2011	Specific focus on maternal health for adolescents
Ministerial directive on maternal death notification	May 10, 2011	Set new policy direction for maternal death review to include criminal investigation and prosecution
Reproductive maternal, new-born and child health sharpened plan for Uganda	2013-2015	Update of policy focus for maternal health to “selective high impact interventions”

Abbreviations: MDG, Millennium Development Goal; RH, reproductive health.

We interviewed 30 key informants and conducted 2 FGDs with selected members of the MoH Technical Working Group for Maternal and Child Health and the CSOs’ Coalition on Maternal Health. The purpose was to triangulate data and generate first-hand experiences on the roles elites played, their interests and how they exercised power in the policy processes. Key informants were identified during document review and, subsequently, through snowball sampling. The inclusion criteria were: (1) being a national policy-maker/actor and, (2) having directly participated in policy-making for maternal health in Uganda between 2000 and 2015 (MDG period). Participants were drawn from all the stakeholder groups acknowledged in the policy documents reviewed (see Table 2). Key informants provided detailed insights on how elite interests and power played out during the policy process while FGDs provided a wider scope of views on the elite interests that influenced decision-making. We used an interview guide in both cases with four broad guiding questions: (1) as one of the participants in making maternal health policies in Uganda between 2000 and 2015 (MDG period), briefly tell me what they entailed. (2) Which other participants/groups do you remember getting involved in maternal health policy-making, and what roles did they play? (3) What were you and other policy-makers trying to achieve in those policies? (4) How did you and those who participated in policy-making for maternal health go about achieving the intentions you have stated above? By answering these questions, participants spoke about their interests and power, and those of other elites who participated in policy-making.

**Table 2:** Categories of Participants by Type of Interviews Conducted

**Category**	**Number Interviews**
HDPs (WHO, UNFPA, World Bank and USAID)	4
Maternal health researchers	2
Consultants on policy development for maternal health	3
MoH officials	5
Retired MoH officials	5
Agencies affiliated to MoH (Uganda Blood Transfusion Services, Health Service Commission, National Medical Stores)	4
Senior politicians (parliament and cabinet)	4
CSO leaders	2
Health professional associations	1
CSO Maternal Health Coalition (FGD)	1 (n = 10)
MoH Maternal and Child Health Technical Working Group (FGD)	1 (n = 8)

Abbreviations: HDPs, health development partners; WHO, World Health Organization; UNFPA, United Nations Population Fund; USAID, United States Agency for International Development; MoH, Ministry of Health; CSO, civil society organization; FGD, focus group discussion.

### Ensuring Rigour

To identify the most relevant documents and study participants, we applied clearly spelt out criteria. Sequencing data collection starting with document review, followed by key informant interviews and concluding with FGDs facilitated triangulation of data and methods. We started with document review to familiarise ourselves with the policies, actors and context. This informed purposive selection of more documents to review and identification of actors to consider as study participants. Document review also informed the sharpening of guiding question and potential areas to probe during the interviews. Iteration between document review and interviews was done to follow up and clarify emerging issues during the research process. To gain rounded insights on the experiences, we interviewed different people at the same level (horizontal interviewing) and across levels (vertical interviewing) within the structures of policy-making. The entire data collection process took four months, allowing saturation in data collection.

### Data Analysis and Interpretation

Data analysis was informed by a constructivist – interpretive paradigm which attempts to generate meanings and explanations about social realities based on participants’ experiences and perceptions.^34,35^ We followed the 3 steps of thematic analysis described in literature^36^ (as recently operationalised in a study in Tanzania^37^). All interviews were audio-recorded and transcribed verbatim. Interview transcripts and filled document review templates were uploaded in Nvivo 12. Based on the study questions, all data were inductively coded line by line to identify the actors, their roles, interest and influences in the policy process. The lead author generated query reports which were independently reviewed by the third, fourth and fifth authors. Descriptive codes and analytic themes were jointly developed by all authors.

**Figure 3:**
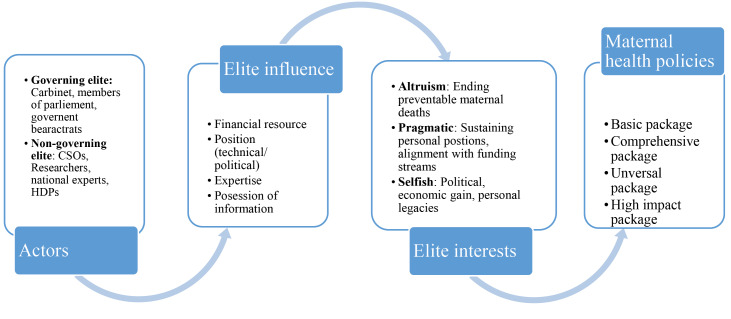
A Step by Step Analysis of Elite Influence in the Policy Process. Abbreviations: CSOs, civil society organizations; HDPs, Health development partners.

We applied the analytic framework by: first, describing the elite group and their different sources of power, second, identifying elite interests of altruism, pragmatism and self-interest as the main analytic themes, and different forms of power applied by the actors to pursue their identified interests during the policy process (Figure 3). Since actors are identifiable with the various policies (they are listed in the acknowledgement sections of policy documents), we made limited and cautious use of explicit examples of policies in the presentation of findings. Instead, emphasis was put on elaborating the elite interests underpinning the maternal health policies we considered and the different forms of power at play.

Interests were analysed as the main themes and described by corresponding subthemes, then linked to the different actors and how they exercised power to influence maternal health policies. Analytic rigour was achieved through inductive thematic saturation, by ensuring that the codes and themes were exhaustive.^38^

## Results

Uganda’s stipulated policy process vests policy-making in central government structures which are predominantly controlled by the policy elites. Although on paper it appears to be a simple process, in reality it is complex as multiple subgroups have unequal access to different sources of power, as well as different interests. Senior MoH officials, some members of the executive, and HDPs had significant influence on maternal health policies. This was because they occupied strategic positions (technical or political), controlled specialised government or global information on maternal health, they had access to financial resources and expertise. Policy elites, acted in a self-interested manner most of the time – especially to win elections, earn economic gain and build personal legacies. In some instances, however, they acted pragmatically by aligning with global maternal health funding streams and pursuing maintenance of organisational programmes or employment. There were a few instances of where policy elites acted altruistically, demonstrating concern to end preventable maternal deaths (achieve policy goals). In the presentation of the detailed results below, we first examine elite composition and power distribution, then present a detailed account of elite interests and influence in the policy processes for maternal health.

### Elite Composition and Distribution of Power

#### Composition of Actors Who Participated in the Policy Process for Maternal Health

The main actors who participated in the policy process for maternal health in Uganda fit the description provided in elite theory. They were located at central level, wielding power and had exclusive access to the policy process. The actors who played roles in the processes were drawn from both the governing elite, those occupying government positions with mandate to make public policies, and the non-governing elite, from outside government structures. The governing elite included: the president, members of cabinet, members of parliament, officials from the ministry of gender and social development, representatives of medical and nursing regulatory bodies, senior and mid-level staff of MoH, researchers from Makerere University Medical school and experienced national level maternal health services providers. The non-governing elite, on the other hand, were: representatives from the private not for profit providers of healthcare, HDPs, members of the Maternal and Child Health Technical Working Group (MCH TWG) and national consultants (Table 3).

**Table 3:** Main Actors Involved in Maternal Health Policies in Uganda in the 2000-2015 (MDG) Period and Their Sources of Power

**Actors**		**Number of Maternal Health Policies in Which Actors Participated (n = 9)**	**Power**
Governing elite with veto power	Minister of finance, planning and economic development	0	Position, control over national budget
	Minister of justice and constitutional affairs	0	Position, expertise on legal matters
	Cabinet secretary	0	Position
	Other members of cabinet	3	Position
	Members of parliament	2	Position, control over national budget
	President	2	Position, control over national budget, Access to privileged information
	Minister of Health	6	Position, expertise on health
	Ministry of gender representatives	2	Position, expertise on gender-related issues
Governing elite controlling policy-making structures	Senior MoH staff	8	Position, expertise on health, control of national health information
Governing elite who play advisory role	Other MoH midlevel staff	8	Expertise in planning and maternal health
	Representatives from National Population Secretariat	3	Control over information on population dynamics, expertise on population matters
	Members of medical and nursing regulatory bodies	5	Expertise
	Researchers from Makerere University Medical School	3	Subject matter expertise, control of research information
	Experienced national level maternal health service providers	3	Expertise in maternal service delivery
Non-governing elite	Private not for profit health representatives	3	Financial resources
	CSOs	5	Information from the grassroots
	HDPs	7	Expertise, information on global developments, position, financial resources
	National consultants	8	Expertise, information
	MoH MCH TWG^a^	2	Expertise, position

Abbreviations: MDG, Millennium Development Goal; CSOs, civil society organizations; HDPs, health development partners; MoH, Ministry of Health; MCH TWG, Maternal and Child Health Technical Working Group. ^a^It is one of the multi-stakeholder committees provided for in the MoH policy development guidance with membership drawn from maternal health professionals, researchers, DPs and CSOs. It provides technical advice to MoH on national maternal health policy and programs.

The Government of Uganda guidelines for policy-making provide for the participation of various groups of actors in the policy process. However, analysis of actors who actually participated in 9 selected maternal health policies, showed that there was variable involvement of the different groups, and some were completely left out of the policy process. The policy process for maternal health was dominated by MoH officials, national consultants and HDPs. Members of the executive only participated in 3 of these policy processes. On average, other governing and none governing policy elite groups participated in 3 out the 9 policies analysed.

### Power Distribution Among Policy Elites

Across all elite groups, power was unevenly distributed. Actors in political positions such as elected national representatives and members of the executive derived significant power from their formal positions. They had powers to appoint senior MoH bureaucrats, appropriate national budgets and approve all government policies. Their power was referred to as veto power – they wielded power to stop the policies at any stage in the process. In practice, however, they were bypassed for most of the policies. The second sub-group included senior bureaucrats in the MoH, National experts (consultants) and HDPs. Senior bureaucrats in the MoH have expertise around maternal health accumulated over many years of professional public service, access to extensive national health information and they occupy strategic positions in the structures that govern the policy process. These actors stewarded policy formulation from inception to completion. National experts (consultants) and HDPs, even though they are not part of the governing elite, had substantial power and access to the structures controlling the policy process. They derived power from technical expertise, latest national and global information, their positions and financial resources to fund policy formulation and implementation. Other members of the governing and non-governing elite mainly had information and expertise. Their participation in the policy process was on invitation by the elites with more power and their ideas were advisory only.

**Figure 4:**
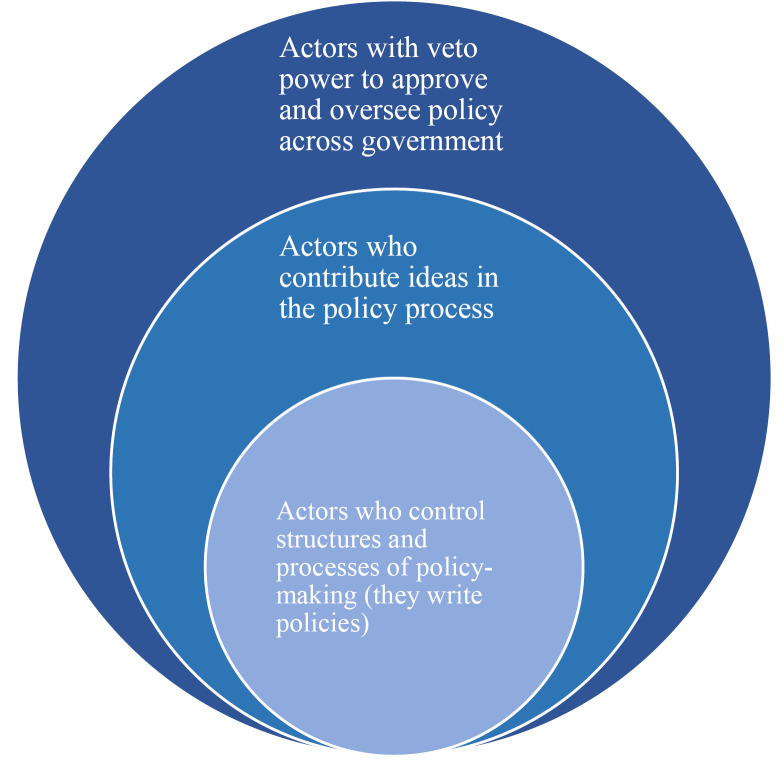
Constituent Subgroups of the Governing Elite Based on Distribution of Power.

From the above analysis of power distribution among policy elites, we unpacked the 2 generic groups described in elite theory and identified 3 elite subgroups (Figure 4). First, a small group with unlimited access and control of the structures and processes for policy-making. This included very senior bureaucrats occupying strategic positions in MoH. They are experienced and generally highly trusted by members of the subgroup with veto power (we explain this later). Through their positions, they derived power to convene the policy processes, setting terms of reference for consultants, determining the actors to invite to participate and clear policy content. HDPs and national consultants were also in this group. HDPs provided technical expertise directly through the secondment of experts or consultants, and funded the policy process and implementation through health sector budget support, vertical programmes and CSO funding. The national consultants designed and wrote the policies to conform to the terms of reference. The second subgroup, comprised of mid-level bureaucrats within MoH, other bureaucrats across government, researchers and other members of the non-governing elite. Their main source of power was expertise and information, warranting their being consulted to contribute ideas in the policy process. The third group comprised of very senior policy actors with the mandate to approve and oversee overall government policy – the majority of whom hold political positions. This last subgroup, generally, should have the final say on the fate of any policy of government and can stop it at any stage, and so, as discussed, they have veto power: the power to block policy.

In practice, in the maternal health policy experiences (Table 3), elites from the inner circle (Figure 4), took the lead throughout the policy process. They wrote maternal health policies and managed the rest of the elites in the policy process. Elites in the middle circle participated on invitation by the members of the first group to contribute their ideas.

Elites in the overarching outer circle only occasionally participated in the policy process directly or through directives, guidance and resolutions. For example, the president and parliament only engaged in 3 out of the 9 processes reviewed. The ministers of finance, planning and economic development, and, justice and constitutional affairs, and the cabinet secretary did not engage in any of the 9 processes (Table 3). Two reasons explain this. First, in some cases, they were cautiously bypassed, as policies were written in the form of programme/project documents, guidelines or strategies – a category of policy documents that does not have to go through all the established government policy processes. Respondents argued that this type of document would not be subject to the formal procedures involved in the formal policy process. Second, some members of the elite group with veto powers actually knew about the policies being developed but preferred to have them as strategies and programme documents. This was to avoid committing government to financially support them, and to avoid political repercussions in case of failure.

*“If you pass them (programmes/projects) as policies, they would require budgetary allocation and parliament can insist that they are implemented through budgetary allocations because they pass the budget. Government (the executive) made sure that it kept quiet to avoid to be put to task because members of parliament are representatives of people, they are stakeholders in these things. When cabinet has not endorsed it, parliament cannot force but it can only advise – which is not enforceable”*(S_P_01).

Breaking down policy elites into clear subgroups helps build a clear understanding of their dynamic interactions in the policy process. It also assists in deciphering elite interests and the exercise of power by the different groups during the policy process.

### Elite Interests Shaping Policies for Maternal Health

As discussed earlier, the elite interests that shaped maternal health policies can be categorised into 3 domains: (*i*) self-interest, especially political and economic gain, (*ii*) pragmatic choices, balancing self-sustenance goals, doing something about maternal mortality and meeting the conditions for programme funding, and, (*iii*) a primary concern for ending preventable maternal death.

### Elite Self-interests

From the interviews, the self-interests pursued by elites were, broadly, private economic and political gain. Political interests were pursued by members of the governing elite holding political positions acquired through elections or political appointment. These interests were threefold: vote-winning, establishing personal legacies and maintaining political appointments.

### Vote Wining

Political elites used their positional influence and control over the national budget to pursue their vote winning interests. They prioritised constructing buildings which could easily be showcased during election campaigns, rather than less tangible provisions like skilled birth attendance, quality of care and health facility functionality. Interventions considered difficult to display were not prioritised. They deflected public scrutiny regarding poor health services onto health workers, referring to them as obstinate and saboteurs of government programmes.

*“There is a time when there was a promise that Health Centre IVs (HC IVs) would be functional, the structures were built they ended up as shells with no human resources, where human resource was deployed, you would find no equipment or you would find a doctor but no anaesthetic specialist. Politicians operated under a cover-up. One time, a maternal death occurred in [facility withheld], politicians claimed the health workers had refused to work on her (the deceased) after she failed to buy drugs and supplies. Later on, I heard that some people (within the political class) were seen ferrying supplies to the facility so that they claim that supplies were available”*(Interview: PA_XPT_01).

Respondents from CSOs (n = 2), MoH (n = 2) and some politicians (n = 2) suggested that politicians developed “policies with broad goals and ambitious targets,” which would not bind them to specific maternal health outcomes. Participants said such policies were written in technical maternal health language to convey “good” intentions while paying lip service to maternal health needs. For example, some respondents questioned policies on free health services for all wondering “*why politicians were not truthful to acknowledge that government could not look after everyone – and encourage citizens to contribute for their health*” (*PA_XPT_01*). An MoH respondent also noted:

*“…it (Health Centre IV) was political. Do you know where these budgets came from? They were manifesto budgets. So, you see where the drive was coming from, there was a certain agenda that has to do with getting votes, not tangible maternal health gains”*(Interview: MOH-STF_IS_01).

Some respondents who played key roles in drafting some of the policies had this to say: “*in some cases, we were ordered to put certain targets- saying you cannot say that all those people will die and we are in charge*” (NC_XPT_01).They set ambitious targets to portray “commitment to ending preventable maternal deaths” even when they knew such targets were difficult to achieve in the circumstances. When we reviewed the targets for reducing maternal mortality which were to be achieved by the various maternal health policies during the MDG period, we found that all of them have to date not been achieved.

### Advancement of Personal Legacies

Some policies were developed to advance the personal legacies of individuals. MoH respondents (n = 2) gave us an insider account of how political appointees exerted their positional power to “push for policies which they would be remembered for” during their tenure at MoH. They normally referred to such policies as* ‘my baby’* – a term used to associate policies with specific individuals. Experts (n = 2) and a politician (n = 1) also shared similar experiences. While they recognised that maternal health benefited in the process, it was also affected by the volatility associated with turnover both among such actors and policy initiatives (despite problems remaining the same). It was observed that in pushing forward their interests, their positions and actions on any issue effectively became policy. Respondents from the MoH referred to such actors as “the policy.” Such policies were usually issued in the form of directives and circulars and were difficult to rescind.

*“If you listen to the ministry of health colleagues, they will tell you that policy was my baby, I had to make sure that it works, now you want to destroy it. They would overpower everyone else, set aside previous policies and come up with their own policies”*(Interview: MoH_RTD_STF-01).

### Maintaining Political Appointments

Political appointees, faced the dilemma of keeping their jobs and delivering on maternal health. Their contracts were subject to approval by members of the elite sub-group with veto power who included senior politicians and bureaucrats. Participants from MoH said that these appointees were preoccupied with retaining their positions by taking care of the interests of their appointing authority. Maternal health followed after “*what puts bread on their dinner tables*” was secured. During the policy process, they used their positions to take care of their personal interests by carefully considering the interests of the elite group with veto power to avoid any confrontation that would jeopardise their appointments. They adjusted their decisions to suit those of their appointing authority. This gave rise to some policies which were difficult to implement or ended up hurting the system. For example, participants referred to a controversial policy directive linked to quality improvement which defined maternal death in part as a crime committed by healthcare providers, subject to criminal investigation and prosecution. Respondents from the FGD with the MCH TWG (comprising of actors from MoH and CSOs) said that front line implementers became reluctant to implement quality improvement processes given the directive, while other health workers resorted to rewriting medical notes in case of a maternal death, to “clean up” the file in case of any potential criminal liability. Some files even disappeared.

### Economic Interests

Participants from CSOs (n = 2), MoH (n = 2) and experts who facilitated some policy processes (n = 2) held the perception that some governing and non-governing elites (some MoH actors and consultants, respectively) were driven by potential monetary gain in relation to the policies developed on maternal health. Using their expertise and positional power, they would seek grant opportunities from among the HDPs. They used maternal health as a flagship programme to attract such grants for activities which on top of maternal health, served their personal economic interests. Respondents said such policies were introduced as projects operated at the MoH headquarters as vertical programmes to maintain central control. They prioritised actions such as meetings, consultations, document production, dissemination, office furnishing, quarterly fuel advances, vehicle procurement and maintenance, and supervision. It was observed that these actions did not sufficiently target maternal mortality. Some respondents referred to the elites who were involved in writing and management of grants for maternal health as “dealers in MoH,” meaning shrewd bureaucrats who used their positions to direct policy in favour of their personal interests. One respondent said;

*“Uganda became money, money, money we were all looking for money. Everybody was looking for a project that would enhance their pockets. Nobody was looking for resources just to merely address maternal death. The leadership at the ministry of health was focusing on how much money they would get (interpretation: there was always a hidden personal agenda for any resource mobilization)”*(Interview: NA_NGE_-01).

Some respondents (n = 2) acknowledged that they were earning income through facilitating policy processes. According to them, the terms of reference for developing programme/project and policy documents were underpinned by more than technical maternal health issues – there were underlying client interests of which they took account when they wrote the documents. During the policy process, their focus was on what they understood as their clients’ interests. Members of the elite group controlling the policy process said that they had to balance technically sound interventions and implementation arrangements with the interests of their clients (such as additional facilitation, travel, office furnishing and new vehicles with generous fuel provision) for the document to be accepted. The actors were driven not by the problem of high maternal mortality but the existence of a grant opportunity and it was the possibility of marketing maternal health as a flagship idea to win the grant that mattered. As one of them said:

*“…whenever it rains in Geneva, we bring out our umbrellas in Kampala to go and work. NC_XPT_02.*Another one added: *It is lucrative, you sit in a hotel, you excite them (donors and government officials) and get paid”*(NC_XPT_02).

### Pragmatic Self-sustenance

As with economic interests, pragmatic self-sustenance also emerged as a shared interest of some members of the governing and non-governing elite who had unlimited access to the structures that controlled the policy process. Within MoH and among some CSOs, maternal health was the mainstay of their programmes and maintaining those programmes over time was important to them. Funding organisations (HDPs), meanwhile, had an international development agenda, but employed local staff to implement that agenda. To maintain their jobs, local staff advanced the goals of their employers by offering development support/funding to MoH and CSOs. Respondents from funders (n = 2) and CSOs (n = 1) noted that the funding organisations would make available grants with specific goals targeting maternal health. CSOs would then develop programmes aligned to the grant and targeting advocacy of maternal health with MoH. On the other hand, MoH would develop a programme/project also aligned to the grant to support its programme. The activities of all groups aligned with the funders’ development support goals whilst also allowing both the MoH and CSOs to attract funds to sustain their maternal health programmes and their local staff members to maintain their jobs. In effect, then, all of them continued to survive by aligning with maternal health as a trending global health agenda - and even when not the initial or underlying concern (Box 1).

**Box 1. Case Study**

One respondent narrated the story of a policy s/he spearheaded which started as the project of a HDP with which s/he was working. S/he said that the HDP was interested in child health, but needed to use maternal health as an entry point for it programme. All the interventions targeted the child while neglecting the mother. S/he said the HDP, provided a huge project grant to start implementation in selected rural areas. They used the project results and the promise of programme funding to influence policy. By the end of the first phase of project implementation, it evolved into a national policy. On reviewing the related policy developments, we confirmed that it started as a project, and was transformed into a policy focusing on women and children even though its primary outcomes were for child health. On further following up the evolutions in this policy, it was observed that it was changed to pay priority attention to maternal health towards the end of the MDG period because of poor health outcomes for children. The maternal health focus was necessary as it was realised that the intended child health goals depended on good maternal health outcomes. The HDP supported all the processes leading to the new policy.

Abbreviations: MDG, Millennium Development Goal; HDP, health development partner.

Some respondents from among the HDPs reported experiences of leveraging the positional power of senior MoH bureaucrats to support drafting of policy documents. They funded high-level national ceremonies with media coverage to obtain endorsement in form of a statement (speech) read by the chief guest (but written for him/her by MoH bureaucrats, sometimes with the input of the HDP) or publicly signing a copy of the documents. One of the HDP respondents said thus:

*“They (government of Uganda) did not decide much, partners came and wrote policies but of course in the presence of MoH officials. Our goals would be documented, endorsed and owned by government as policy. All these processes were facilitated by partners through funding, technical support and representatives of government would only sign documents”*(Interview HDP_FH_01).

Respondents noted that given the pursuit of funding, maternal health programmes and policies often mimicked global imperatives (see Table 4) rather than being adapted to the national context. Several policies and programmes consequently followed after global commitments rather than the changes in the national dynamics of maternal mortality.

**Table 4:** National Policies and Related Global Imperatives in the MDG Period

**Policy Documents**	**Period**	**Global Imperatives**
National health policy	1999-2009	Follow up on primary healthcare
The national policy guidelines and service standards for reproductive health services	2001-2005	ICPD
The national policy guidelines and service standards for sexual and reproductive health and rights	2006	ICPD and MDGs
Roadmap for accelerating the reduction of maternal and neonatal mortality and morbidity in Uganda	2007-2015	Fast-tracking MDGs 4 and 5
Second national health policy	2010 - to date	Alignment with MDGs
Reproductive maternal, new-born and child health sharpened plan for Uganda	2013-2015	Every woman every child

Abbreviations: MDG, Millennium Development Goal; ICPD, International Conference on Population and Development.

*“Following his return from heads of state meeting in New York in 2005, [name excluded] convened a meeting on the state of maternal and newborn health. He tasked the Ministry of Health to develop a master plan to address the issue of high maternal mortality. Consequently, the Ministry of Health, with other sectors and partners, have renewed their commitment to addressing maternal health issues and has developed a Road Map for Reduction of maternal and new-born morbidity and mortality in Uganda”*(Extract from rationale. Roadmap. p. 22).

One of the consultants who facilitated some of the policy processes said:

*“That (changing global policies) is what kept us (Uganda) in motion. When it came to policies, we would introduce a policy, then [global actor] would come up with another one then we would move on to the next before we could realize the results another one would come up. It was more of an issue of review, revise, abandon and the cycle continued. But we also knew that it was not about us being creative to change and save mothers from postpartum haemorrhage, there are very many factors that come to play”*(Interview: NC_NO_01).

### Altruistic Concern to End Preventable Maternal Death

Across all elite groups there were actors with concern for ending preventable maternal death, even those who could not influence the policy process. Some HDPs (n = 2) were the only ones who succeeded in influencing maternal health policies in line with the goal of reducing maternal mortality. One of the HDPs said that through financial resources, technical expertise and global positioning they had access to both policy elites with veto power and those controlling the structures of the policy processes. They influenced maternal health policies towards the goal of reducing maternal mortality by conditioning grants towards policies addressing Emergency Obstetric Care and increased access. Members of parliament not in executive positions, mid-level officials of MoH, members of professional associations and CSOs observed that although they had good ideas, they could not get actors in control of the policy process to take up their ideas. Participants from MoH in midlevel positions (n = 2) noted that they had concern for ending preventable maternal deaths but did not have enough positional power to direct policies to this goal. They said they were left to work within the policy priorities set by the powerful elites even though they were not primarily targeting maternal mortality. Other participants who retired from service with MoH (n = 2) noted the same. Some members of parliament (n = 3) expressed frustration that they were unsuccessful in their initiatives to influence other elites to support policy actions focused on reducing maternal death. They attributed this to a lack of sufficient positional power to exert influence on the elites with veto power towards reducing maternal mortality. CSOs under the leadership of their peers with legal expertise also attempted to influence the policy process toward ending preventable maternal death through a 2011 court case (Constitutional Petition No. 16). They wanted the courts to declare preventable maternal deaths a violation of the right to life by the state. The aim of the petitioners was to secure a court decision-making it mandatory for government to ensure that every pregnant woman accesses lifesaving interventions when they need them Court ruled in their favour.

## Discussion

This paper provides a detailed examination of the elite interests and power dynamics that shaped Uganda’s maternal health policy-making in the period 2000-2015. It illustrates how multiple elite groups exercised power during the policy process to influence maternal health policies towards their diverse and competing interests. We contend that power in its different forms instigated a reconfiguration of the elite groups as they engaged in the policy process. In contrast to the processes envisaged in Uganda’s formal guidance for policy-making, in practice actors controlling the structures for policy-making had more power than the rest. Elite groups with more power (position, expertise and financial resources) were more self-interested and influential while altruistic interests tended to be pursued mostly by the less powerful elites (and some HDPs). In the end, policies persistently yielded incidental benefits for maternal health without achieving their set targets.

### Elite Formation and Influence in the Policy Process

Centralisation of the policy process with exclusive participation of elites is one of the challenges of public policy management in Uganda. Central policy elites are organised in different formations with varying influence. The control of policy processes in Uganda by a few bureaucrats occupying strategic positions in government departments loyal to political elites continues.^39,40^ Highly placed bureaucrats tend to have more authority, trust by the political elite and control over the policy processes and moderate participation of all actors. Similar influence was exercised by bureaucrats in Burkina Faso during the policy process for integrated community case management.^41^ HDPs operating in Uganda also have access to and influence on the structures that control the policy process through their consistent participation, technical and financial support.^1,39,42^ The combined influence of key MoH bureaucrats and donors is also visible in the Ugandan policy processes for human resources for health, disease prevention and family planning programmes.^2^ The experience of the abolition of user fees in Uganda shows that elites other than donors, political and bureaucratic actors have no significant influence on the policy process.^43^ Our findings show that the less powerful elites merely contribute ideas but cannot influence the policy process. CSOs, researchers, mid-level bureaucrats and professional associations commonly fall into this category, and do not influence final decisions.^44^ Even among the groups with significant formal power, such as the Ugandan elite group with veto power, not all had influence over policy processes. A key finding from this study is that the elites who influenced policies often acted in self-interest, whilst other actors had limited influence on the policy process.

### Elite Interests and the Exercise of Power in the Policy Process

This study shows that elites who participated in the policy processes for maternal health in Uganda were driven by multiple interests. These ranged from political, economic, the search for feasible policies and the need to end preventable maternal death. The study also advances our understanding of the exercise of power by elites to promote these interests during the policy process.^45^

Vote wining is the main political interest that has been written about in Uganda. This happens as political and bureaucratic elites seek to consolidate and maintain their positions for personal gain.^40^ As observed in other analyses, productive sector policy reforms which were likely to negatively affect votes in Uganda were dropped in favour of those that enhanced opportunities for election victory.46 Similar to the findings of this study, even when vote winning has sometimes influenced policies responding to perceived public interests, such policies tend to pursue short-term visible results.^47^ The actors ensure that their policies can clearly be linked to them by the voters at the time of seeking political support. Similarly, this study shows that the political elite put in place policies with interventions that were immediately visible and could be linked to their legacies; but with broad enough goals to accommodate their interests.

In addition to political interests, the findings of this study reflect earlier Ugandan analyses which showed that in the 1990s economic interests significantly influenced health policy.^1^ Other analyses show that technocrats in the MoH derived personal economic benefit from donor funded policy reforms for malaria, Tuberculosis and HIV and AIDS. This was treated as their reward for their loyalty to the political elite.^46^ Similarly, for maternal health some officials of the MoH were reported to be engaged in donor driven policy reforms for personal economic gain. This gives room for donors to impose policies in furtherance of their global agendas.^1,39,40,48^

Since maternal health policy processes involved competing actors with diverse interests^2,49^ actors sometimes pursued feasible alternatives (pragmatic interests). In some cases, policies were calibrated to fit donor conditionalities.^1^ Political feasibility was also reported as one of the drivers for policy reforms in Uganda’s productive sector, requiring balance between addressing the actual problem and political goals.^46^ In this study, elites were mostly adjusting to fit with donor funding streams. Closely relate to the above, some actors among the bureaucratic and political elite also sought to reduce maternal mortality, as their primary interest. Such altruistic behaviours by some elite groups such as professionals who engage in policy processes seeking to improve standards of care, have also been reported elsewhere in Sub-Saharan Africa.^29,48^

As noted from the study findings, influence over a specific policy process depends on the combination of interests and power, as well as the balance of power among elite groups.^48,50^ This study shows that the MoH technocrats leading maternal health policy processes understood this well. They effectively worked around the Ugandan elites with formal veto power and the remaining actors in the various policy processes. The technocrats took account of the interests of those they perceived to be more powerful and bypassed the formal policy processes by introducing policies through programme documents which would not undergo formal policy scrutiny.

Given that navigating and serving the personal interests of the powerful elite dominated maternal health policy processes, this study points to the fact that such processes gave rise to “public” policies that did not necessarily wholly address a public health problem or serve public interests. This contradicts the normative assumption that elites holding public positions serve public interests.^29,51^ Policy elites often seek to preserve themselves as a group^4,52^ and may take into consideration public interests if their positions become threatened.^6^ While the public goal and targets of the various maternal health policies in Uganda aimed to reduce maternal mortality, the multiple personal interests that drove them were not primarily intended to reduce maternal mortality. Consequently, none of the policies across the fifteen-year period examined achieved their written targets on the public problem of high maternal mortality. Similarly, public health policies on alcohol control in Lesotho, Uganda, Malawi and Botswana were heavily influenced by the business interest of the alcohol industries and neglected the public interests of reducing harmful alcohol consumption and its related health problems.^53^

### Limitations

This paper is informed by the perspectives of national policy-makers who participated in the maternal health policy process during the MDG period. Their views inform our conclusions about the influence of Uganda’s policy elites on maternal health policies. Given that it derives from the elites who directly participated in the policy processes, the study brings first-hand experiences of what transpired in the policy processes. Analytic rigour was maintained by triangulating data across stakeholder groups of the governing and non-governing elite, and with policy documents covering the entire MDG period.

## Conclusion

This study shows that Uganda’s maternal health policies across the MDG period were largely influenced by elite personal interests and only in a few instances reducing maternal mortality. As a consequence, these policies yielded incidental benefit for maternal health. This situation emanates from the formal guidance for policy-making in Uganda which gives elites control over the policy process and so, the latitude to determine policy. Citizens are officially excluded from direct involvement in policy-making by these guidelines and this may explain why public interest did not significantly drive maternal policies.

Addressing maternal health challenges will require stronger mechanisms to ensure that public officials do not infuse policies with their interests. This entails re-engineering the policy process and opening up space for meaningful percolation of ideas from the frontline and the public – and holding the elite accountable for both the outcomes of the policy process and implementation.

## Acknowledgements

We are grateful to the national policy-makers in Uganda who participated in this study. We were also assisted by the Director General of Health Services to convene the MNCH Technical Working Group. We are grateful for the assistance we received from the Centre for Health Human Rights and Development as a secretariat for the CSO coalition on MCH to convene a focus group interview with its members. We acknowledge Professor Jeremy Shiffman from Department of International Health and School of Advanced International Studies, Johns Hopkins University for his initial guidance and feedback on the draft manuscript. We acknowledge the German Academic Exchange Service for doctoral training scholarship and the Erasmus *Plus* mobility programme of Nottingham Trent University for the writing residency support.

## Ethical issues

This study was approved by the Higher Degrees Research and Ethics Committee of the School of Public Health, Makerere University and cleared by Uganda National Council for Science and Technology (Ref: SS 4484). We obtained informed consent from all participants after verbal explanation and reading and signing the consent form. Key informants were interviewed on appointment at their convenience. Participants of the FGDs with the CSO coalition on maternal health were invited through their secretariat while those for the MoH Technical Working Group on Maternal and Child Health were invited through the Director General of Health Services on the advice of the chairperson of the technical work group. Invitation letters were sent two weeks in advance with an abstract of the study protocol. Interviews and quotes were anonymised using respondent numbers.

## Competing interests

Authors declare that they have no competing interests.

## Authors’ contributions

MM, FS, and SNK conceptualized and designed the study. MM participated in data collection. MM, FS, SNK, LG, and MSK conceptualised the paper. All authors participated in data analysis, MM drafted the paper with guidance from FS, SNK, LG, and MSK. All authors reviewed the draft manuscript and provided critical feedback with recommendations. All authors reviewed and approved the final manuscript.

## Disclaimer

The views expressed in this article are for the authors and not the position of the institutions of affiliation or the funders.

## Funding

This paper has been funded through the Health Policy Analysis Fellowship programme, supported by the Alliance for Health Policy and Systems Research, Switzerland. The research work was also funded by the SPEED Project – a project at Makerere University School of Public Health focusing on evidence generation to inform health policy-making in Uganda.

## Authors’ affiliations

^1^Department of Health Policy, Planning and Management, School of Public Health, College of Health Sciences, Makerere University, Kampala, Uganda. ^2^Health Policy and Systems Division, School of Public Health and Family Medicine, University of Cape Town, Cape Town, South Africa. ^3^Department of Global Health and Development, London School of Hygiene and Tropical Medicine, London, UK.

## Key Messages

**Implications for policy makers**

- Policy elites are a fluid group with often unwritten interests ranging from self-interest, pragmatism to altruism – all of which should be keenly observed and regulated during the policy process to achieve public interest policy goals.
- Since elites and their actions can sometimes be driven by unwritten personal interests which are contrary to written policy goals, pursuing the public interest requires recognising and seeking to limit the influence of other interests over policy-making.
- Achieving accelerated reduction in maternal mortality in the post Millennium Development Goal (MDG) era will require reforms that will refocus policy actions back to the main causes of high maternal mortality backed by stronger mechanisms for holding policy elites accountable for their written policy goals.

**Implications for public**

Examining the elite interests that drive policy actions is central to our understanding of why certain policies work or fail. These interests may not always be manifest. Efforts to explain why many low- and middle-income countries missed targets for Millennium Development Goal five (MDG 5) and continue to experience high maternal mortality have not paid adequate attention to the role of elite interests in policy failure. This paper draws the attention of policy practitioners and scholars to the interests that underlie policies, to inform meaningful policy development.
